# Supplementary material for: ARHGAP10 is a novel microtubule-associated protein that regulates the resorption activity of osteoclasts
Source: J Biol Chem. 2025 Aug 30;301(10):110668. doi: 10.1016/j.jbc.2025.110668 (PMC12509757; doi:10.1016/j.jbc.2025.110668)
Supplement: Supporting information [file mmc3.pdf]

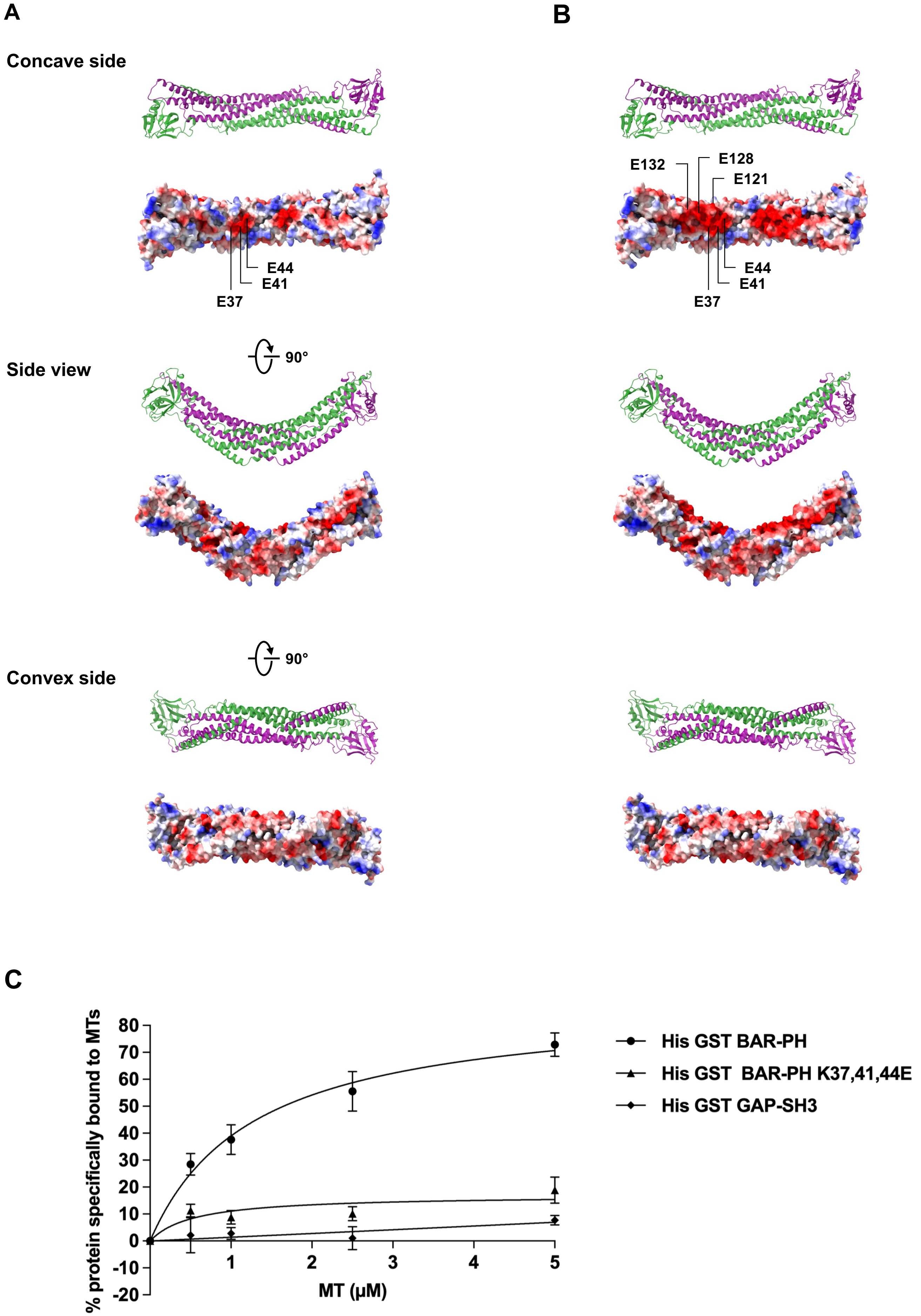

A
B
Concave side
E132
E128
E121
E44
E41
E37
Side view
90°
Convex side
C
% protein specifically bound to MTs
MT (µM)
His GST BAR-PH
His GST BAR-PH K37,41,44E
His GST GAP-SH3

Figure S1

D

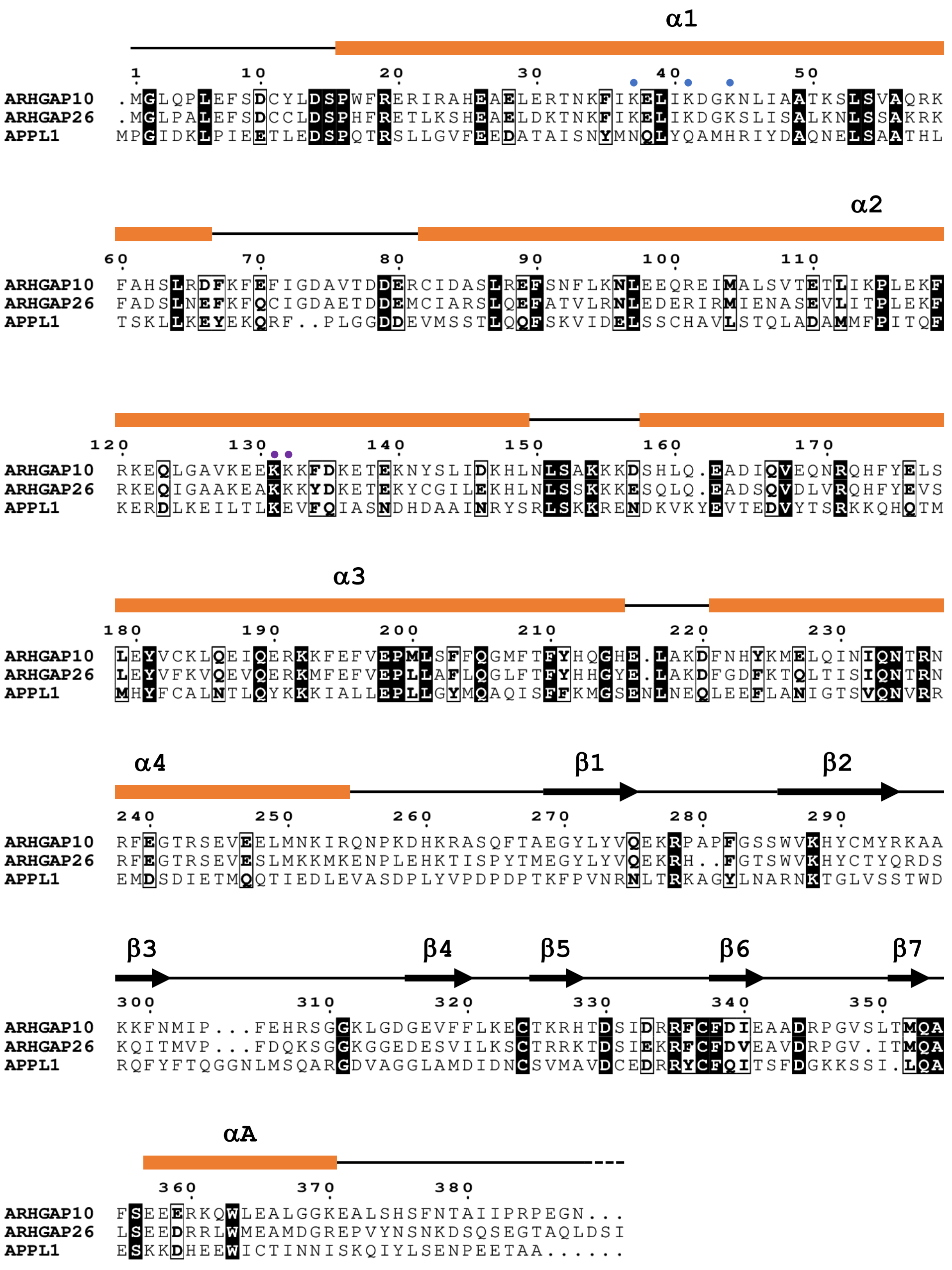

α1
1 10 20 30 40 50
ARHGAP10 .MGLQPLEFSDCYLDSPWFRERIRAHEAELERTNKFIKELIKDGKNLIAATKSLSVAQRK
ARHGAP26 .MGLPALEFSDCCLDSPHFRETLKSHEAELDKTNKFIKELIKDGKSLISALKNLSSAKRK
APPL1 MPGIDKLPIEETLEDSPQTRSLLGVFEEDATAISNYMNQLYQAMHRIYDAQNELSAATHL
α2
60 70 80 90 100 110
ARHGAP10 FAHSLRDFKFEFIGDAVTDDERCIDASLREFSNFLKNLEEQREIMALSVTETLIKPLEKF
ARHGAP26 FADSLNEFKFQCIGDAETDDEMCIARSLQEFATVLRNLEDERIRMIENASEVLITPLEKF
APPL1 TSKLLKEYEKQRF..PLGGDDEVMSSTLQQFSKVIDELSSCHAVLSTQLADAMMFPITQF
120 130 140 150 160 170
ARHGAP10 RKEQLGAVKEEKKKFDKETEKNYSLIDKHLNLSAKKKDSHLQ.EADIQVEQNRQHFYELS
ARHGAP26 RKEQIGAAKEAKKKYDKETEKYCGILEKHLNLSSKKKESQLQ.EADSQVDLVRQHFYEVS
APPL1 KERDLKEILTLKEVFQIASNDHDAAINRYSRLSKKRENDKVKYEVTEDVYTSRKKQHQTM
α3
180 190 200 210 220 230
ARHGAP10 LEYVCKLQEIQERKKFEFVEPMLSFFQGMFTFYHQGHE.LAKDFNHYKMELQINIQNTRN
ARHGAP26 LEYVFKVQEVQERKMFEFVEPLLAFLQGLFTFYHHGYE.LAKDFGDFKTQLTISIQNTRN
APPL1 MHYFCALNTLQYKKIALLEPLLGYMQAQISFFKMGSENLNEQLEEFLANIGTSVQNVRR
α4 β1 β2
240 250 260 270 280 290
ARHGAP10 RFEGTRSEVEELMNKIRQNPKDHKRASQFTAEGYLYVQEKRPAPFGSSWVKHYCMYRKAA
ARHGAP26 RFEGTRSEVESLMKKMKENPLEHKTISPYTMEGYLYVQEKRH..FGTSWVKHYCTYQRDS
APPL1 EMDSDIETMQQTIEDLEVASDPLYVPDPDPTKFPVNRNLTRKAGYLNARNKTGLVSSTWD
β3 β4 β5 β6 β7
300 310 320 330 340 350
ARHGAP10 KKFNMIP...FEHRSGGKLGDGEVFFLKECTKRHTDSIDRRFCFDIEAADRPGVSLTMQA
ARHGAP26 KQITMVP...FDQKSGGKGGEDESVILKSCTRRKTDSIEKRFCFDVEAVDRPGV.ITMQA
APPL1 RQFYFTQGGNLMSQARGDVAGGLAMDIDNCSVMAVDCEDRRYCFQITSFDGKKSSI.LQA
αA
360 370 380
ARHGAP10 FSEEERKQWLEALGGKEALSHSFNTAIIPRPEGN...
ARHGAP26 LSEEDRRLWMEAMDGREPVYNSNKDSQSEGTAQLDSI
APPL1 ESKKDHEEWICTINNISKQIYLSENPEETAA......

Figure S1

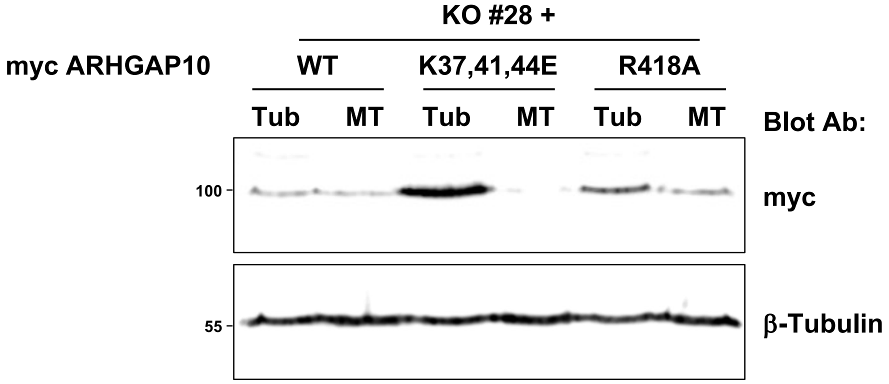
KO #28 +
myc ARHGAP10
WT
K37,41,44E
R418A
Tub
MT
Tub
MT
Tub
MT
Blot Ab:
100
myc
55
β-Tubulin

Figure S2

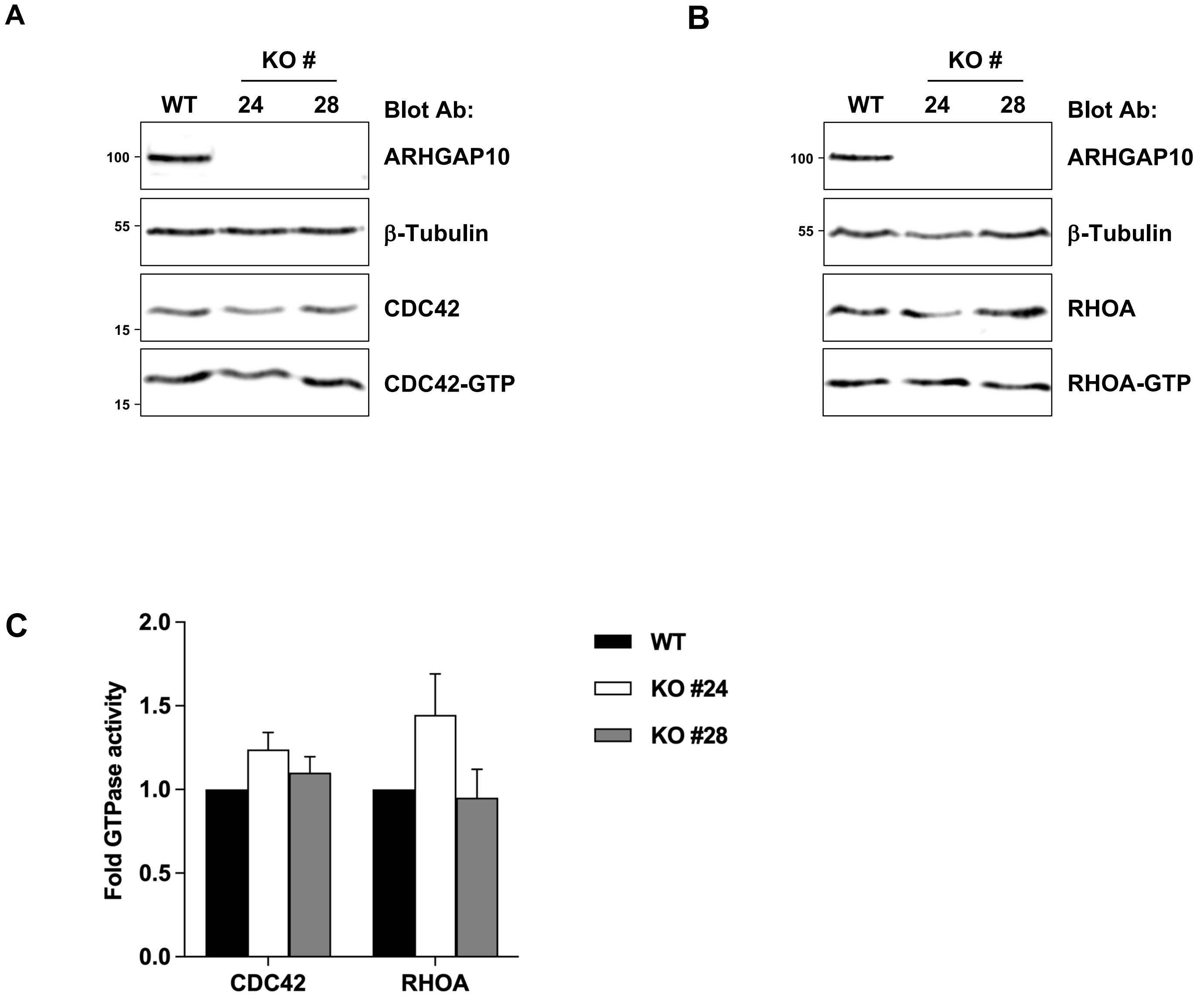
A
KO #
WT
24
28
Blot Ab:
100
ARHGAP10
55
β-Tubulin
CDC42
15
CDC42-GTP
15
B
KO #
WT
24
28
Blot Ab:
100
ARHGAP10
55
β-Tubulin
RHOA
RHOA-GTP
C
Fold GTPase activity
2.0
1.5
1.0
0.5
0.0
CDC42
RHOA
WT
KO #24
KO #28

Figure S3

LEGENDS OF SUPPORTING INFORMATION

Figure S1: Effect of K37,41,44E mutation on structure and in vitro microtubule binding of ARHGAP10 BAR-PH domain.

(A) Structural model of ARHGAP10 BAR-PH K37,41,44E mutant. The BAR-PH domain (1-388) of ARHGAP10 was modelized as in fig. 2A. The corresponding electrostatic surface of each side is represented bellow. Note that introduced mutations do not significantly affect the structure of the BAR-PH domain but the impact of indicated mutations on the charge is obvious mostly in the concave side. **(B)** Structural model of ARHGAP10 BAR-PH K37,41,44E and K121,128,132E mutant. This mutant was modelized and represented as in (A). **(C)** Graph quantifying the microtubule co-sedimentation of His GST BAR-PH K37,41,44E (n = 5, ± SEM) along with the curves corresponding to His GST BAR-PH and His GST GAP-SH3 presented in fig. 1D. **(D)** Sequence alignment of human ARHGAP10, ARHGAP26 and APPL1. The secondary structure elements are indicated. Conserved residues are boxed, identical residues are highlighted by black background and similar residues are bold. Blue and purple dots respectively show residues involved in microtubule binding and in sensing membrane curvature, inducing membrane tubulation, binding tubules.

Figure S2: Microtubule association of exogenous ARHGAP10.

Representative immunoblot analysis of lysates from Arhgap10 KO #28 osteoclast expressing myc-tagged ARHGAP10 WT, K37,41,44E or R418A subjected to tubulin/MT fractionation. Note that the association of ARHGAP10 with MT fraction is significantly inhibited by K37,41,44E mutations but not R418A mutation.

Figure S3: Global CDC42 and RHOA activities are not affected by ARHGAP10 depletion.

(A) Representative immunoblot analysis of active CDC42 pull-down using the GTPase binding domain of N-WASP from WT and Arhgap10 KO osteoclasts. β-tubulin is used as a loading control and osteoclast differentiation is evaluated by mature CtsK. **(B)** Representative immunoblot analysis of active RHOA pull-down using the RHO binding domain of Rhotekin from same cells as in (A). **(C)** Graph representing fold changes in GTPase activity of osteoclast expressing (WT) or not ARHGAP10 (KO #24 or 28) normalized to the activity in control cells (WT) (n = 4 for CDC42 or 3 for RHOA, ± SEM).

Movies S1 and S2.

Live imaging of LifeAct-mCherry expressing WT (movie S1) or KO Arhgap10 #24 (movie S2) osteoclasts. Time is indicated as hour:min and scale bar is 20 µm.
